# Prognostic and immunological significance of metastasis-associated protein 3 in patients with thymic epithelial tumors

**DOI:** 10.1007/s12672-024-01066-1

**Published:** 2024-06-09

**Authors:** Jinping Li, Zhenyan Deng, Yu Liu, Jiamin Jin, Chichu Xie, Jinfeng Gan

**Affiliations:** 1 Department of Histology and Embryology, School of Preclinical Medicine, Guilin Medical University, Guilin, China; 2 Department of Clinical Laboratory, Guilin Hospital of the Second Xiangya Hospital CSU, Guilin, China; 3 Guangxi Key Laboratory of Tumor Immunology and Microenvironmental Regulation, Guilin Medical University, Guilin, China; 4 Guangxi Health Commission Key Laboratory of Tumor Immunology and Receptor-Targeted Drug Basic Research, Guilin Medical University, Guilin, China; 5 Clinical Research Center, The First Dongguan Affiliated Hospital, Guangdong Medical University, Dongguan, China

**Keywords:** MTA3, Thymic epithelial tumors, Tumor microenvironment, Immune infiltration, Prognosis

## Abstract

**Background:**

Immune checkpoint inhibitors have shown promising anticancer activity and have recently been proposed as a therapy for thymic epithelial tumors (TETs); however, this treatment is only effective for a subgroup of TET patients. Thus, this study aims to identify the potential genes implicated in the regulation of cancer immunity in TETs.

**Methods:**

The TETs RNA-seq and clinical data were obtained from The Cancer Genome Atlas (TCGA) database. The clinical significance of the tumor microenvironment (TME) in TETs was evaluated. Weighted gene coexpression network analysis (WGCNA) was used to identify the immune response-related hub genes. The expression of metastasis-associated protein 3 (MTA3) in TETs was investigated in public datasets and a patient cohort. Kaplan‒Meier curves were generated to analyze the prognostic value of various factors. The Tumor Immune Estimation Resource (TIMER2.0) was used to estimate the relevance of MTA3 to immune cell infiltration. Gene set enrichment analysis (GSEA) and pathway enrichment analysis were applied to explore the MTA3-related pathways.

**Results:**

The TME was found to be clinically significant in TETs. Moreover, MTA3 was identified as a key gene associated with the immune score, and lower MTA3 expression was linked to poor TME and reduced cytotoxic activity in TETs. Furthermore, MTA3 was found to be deregulated in TETs, predictive of poor prognosis. MTA3 was also significantly associated with the infiltration levels of various immune cell types and highly correlated with their corresponding markers. Notably, MTA3 was positively associated with various immune response pathways.

**Conclusion:**

MTA3 is clinically significant in TETs and correlated with immune cell infiltration. Thus, MTA3 might be a biomarker for predicting the prognosis and immune status of TET patients.

**Supplementary Information:**

The online version contains supplementary material available at 10.1007/s12672-024-01066-1.

## Introduction

Thymic epithelial tumors (TETs) arise from thymus gland epithelial cells and are the most common tumors in the anterior mediastinum, with an annual incidence of 0.13–0.32/100,000 worldwide [1, 2]. Thymoma and thymic carcinoma are the two most common pathological subgroups of TETs [2, 3]. TETs are classified as malignant tumors because all major subtypes can be aggressive [3]. TETs are primarily treated surgically; complete surgical resection results in a favorable prognosis [4]. However, there are few therapeutic options for relapsed or refractory TETs [5]. Immune checkpoint inhibitors have recently been proposed as treatments for TETs [6, 7]; however, the response rates are below 30% [6, 7]. Therefore, exploring the potential genes involved in the regulation of the immune response in TETs is critical.

Metastasis-associated proteins (MTAs), including MTA1, MTA2, and MTA3, regulate multiple cellular functions by participating in the chromatin remodeling of target genes via association with the nucleosome remodeling deacetylase (NuRD) complex [8]. MTA1 and MTA2 have been extensively studied [8]. In contrast, studies focusing on MTA3 are relatively limited [8]. MTA3 plays a role in various human cancers [9–15]; it is downregulated in, for example, esophageal squamous cell carcinoma [9], gastroesophageal junction adenocarcinoma [16], breast cancer [11], tongue carcinoma [15], colorectal cancer [17], and glioma [14]. Moreover, decreased MTA3 expression is associated with poor patient prognosis in many cancers [9, 14–17]. Functionally, MTA3 can inhibit epithelial–mesenchymal transition (EMT) by directly suppressing Snail transcription by forming a distinct complex with Mi-2/NuRD [18]. Recently, MTA3 has been shown to suppress cancer cell stemness and metastasis by inhibiting the SRY-box transcription factor 2 overlapping transcript (SOX2OT)/SOX2 axis [9]. In addition to its role in cancer, MTA3 has been linked to the regulation of germinal center B-cell differentiation [19–21] and CD4 T cell fate and function via interactions with B-cell CLL/lymphoma 6 (Bcl6) [22], suggesting a role for MTA3 in immune response regulation. However, the relationship between MTA3 and tumor immunity and its role in TETs remains unclear.

Here, we identified MTA3 as a key gene associated with the immune score and investigated its clinical significance and role in the tumor microenvironment (TME) in TETs and the pathways in which it may be involved. We found that MTA3 was associated with TET patients’ clinical outcomes and immune activity. Thus, MTA3 is a potential biomarker for predicting the prognosis and immune status of patients with TETs.

## Materials and methods

### Clinical samples and immunohistochemistry analysis

A tissue microarray (TMA) consisting of 56 thymomas, 18 thymic carcinomas, and 7 squamous cell carcinomas was obtained from Shanghai YEPCOME Biotechnology Company (Shanghai, China). Immunohistochemistry analysis (IHC) was conducted as previously described [23]. Briefly, the TMA was processed and incubated with the primary antibody anti-MTA3 (Proteintech, #14682-1-AP), followed by incubation with a secondary antibody before visualization with 3,3’-diaminobenzidine (DAB) and counterstaining with hematoxylin. Two independent investigators evaluated the immunohistochemical results. The staining intensity of the cells was graded as follows: negative (0), “weak” (1), “moderate” (2), and “strong” (3). The positively stained cells were scored as follows: “0%” (0), “1–25%” (1), “26–50%” (2), “51–75%” (3), and “ > 75%” (4). The IHC score was calculated as follows: intensity score × proportion score.

### Data acquisition

TET mRNA-seq expression profiles and clinical data were obtained from The Cancer Genome Atlas (TCGA) via the National Cancer Institute (NCI) Genomic Data Commons (https://gdc.cancer.gov). The mRNA expression of MTA3 in thymic tumors was investigated in the Gene Expression Omnibus (GEO; www.ncbi.nlm.nih.gov/geo) [24] datasets GSE79978 [25] and GSE177522 [26]. The relationship between MTA3 expression and immune infiltration in TETs was validated in the GEO dataset GSE29695 [27].

### Weighted gene coexpression network analysis and univariate analysis

The TCGA-TET cohort was divided into immune score^High^ and immune score^Low^ groups according to the median immune score. Differentially expressed genes (DEGs) with |Log_2_ (fold change)|> 0.585 and adj. *P* value < 0.05 was identified using the “limma” R package. The DEGs were further subjected to weighted gene coexpression network analysis (WGCNA) to construct a scale-free coexpression network using the “WGCNA” R package. The hub genes in the most relevant modules were identified according to module membership (MM) > 0.9 and gene significance (GS) scores > 0.5. Univariate analysis was subsequently conducted to investigate the relationship between hub genes and TET patient overall survival rates. The mRNA expression of the indicated hub genes in different single-cell clusters in the thymus was investigated using the Human Protein Atlas database (https://www.proteinatlas.org/) [28, 29]. The mRNA expression of the indicated hub genes in various human tissues was investigated using the PaGenBase database (http://bioinf.xmu.edu.cn/PaGenBase/index.jsp) [30].

### Pathway enrichment analysis

The genes that were positively correlated with MTA3 in the TCGA-TET cohort (R ≥ 0.7) were identified using the UALCAN database (https://ualcan.path.uab.edu/index.html) [31]. The genes were subsequently analyzed for BIOCARTA pathway enrichment using the Database for Annotation, Visualization, and Integrated Discovery (DAVID) database (https://david.ncifcrf.gov/). The R package “ggplot2” was used to visualize the enriched pathways.

### Immune infiltration analysis

The correlations between MTA3 expression and the infiltration levels of B cells, CD4^+^ T cells, CD8^+^ T cells, neutrophils, macrophages, myeloid dendritic cells, and cancer-associated fibroblasts (CAFs) were examined with the Tumor Immune Estimation Resource (TIMER2.0; http://timer.comp-genomics.org) [32–34]. The TIMER2.0 database was used to download the infiltration levels of six immune cell types in the TCGA-TET cohort estimated by the TIMER method, as well as the immune, stromal, and microenvironment scores determined by the xCell method [35], and the infiltration level of CAFs and the cytotoxicity score evaluated by the Microenvironment Cell Populations-counter (MCP-counter) method [36]. The relationship between MTA3 and immune cell gene markers was analyzed using the TIMER database (https://cistrome.shinyapps.io/timer/) [33, 34]. For the GSE29695 cohort, immune infiltration was estimated using the MCP-counter method, and immune and stromal scores were computed by the ESTIMATE method [37]. Then the association between MTA3 and immune infiltration, immune score, and stromal score was analyzed, respectively.

### Survival analysis

The 118 TET patients from the TCGA cohort who had follow-up data available were divided into two groups according to median MTA3 expression value or immune, stromal, microenvironment, cytotoxicity scores. The impact of these variables on the overall survival rates of TET patients was subsequently analyzed via Kaplan‒Meier curves.

### Gene set enrichment analysis

The mRNA profiles of 118 TCGA-TET patients were divided into MTA3^High^ and MTA3^Low^ groups according to the median expression value of MTA3 and then processed and analyzed by gene set enrichment analysis (GSEA) as previously described [9, 38].

### Statistical analyses

SPSS 17.0 software (SPSS, Inc., USA) was used for the statistical analyses. Student’s *t*-test or Mann–Whitney test was used to investigate the difference between two data sets as appropriate, and one-way ANOVA was used to analyze differences among more than two groups. The χ^2^ test was used to assess the association between MTA3 expression and clinical factors. Pearson’s or Spearman’s correlation coefficient was used to evaluate the associations between MTA3 expression and various variables, where appropriate. A *P* value < 0.05 was considered to indicate significance.

## Results

### TME is associated with clinical characteristics in TET patients

**Fig. 1:**
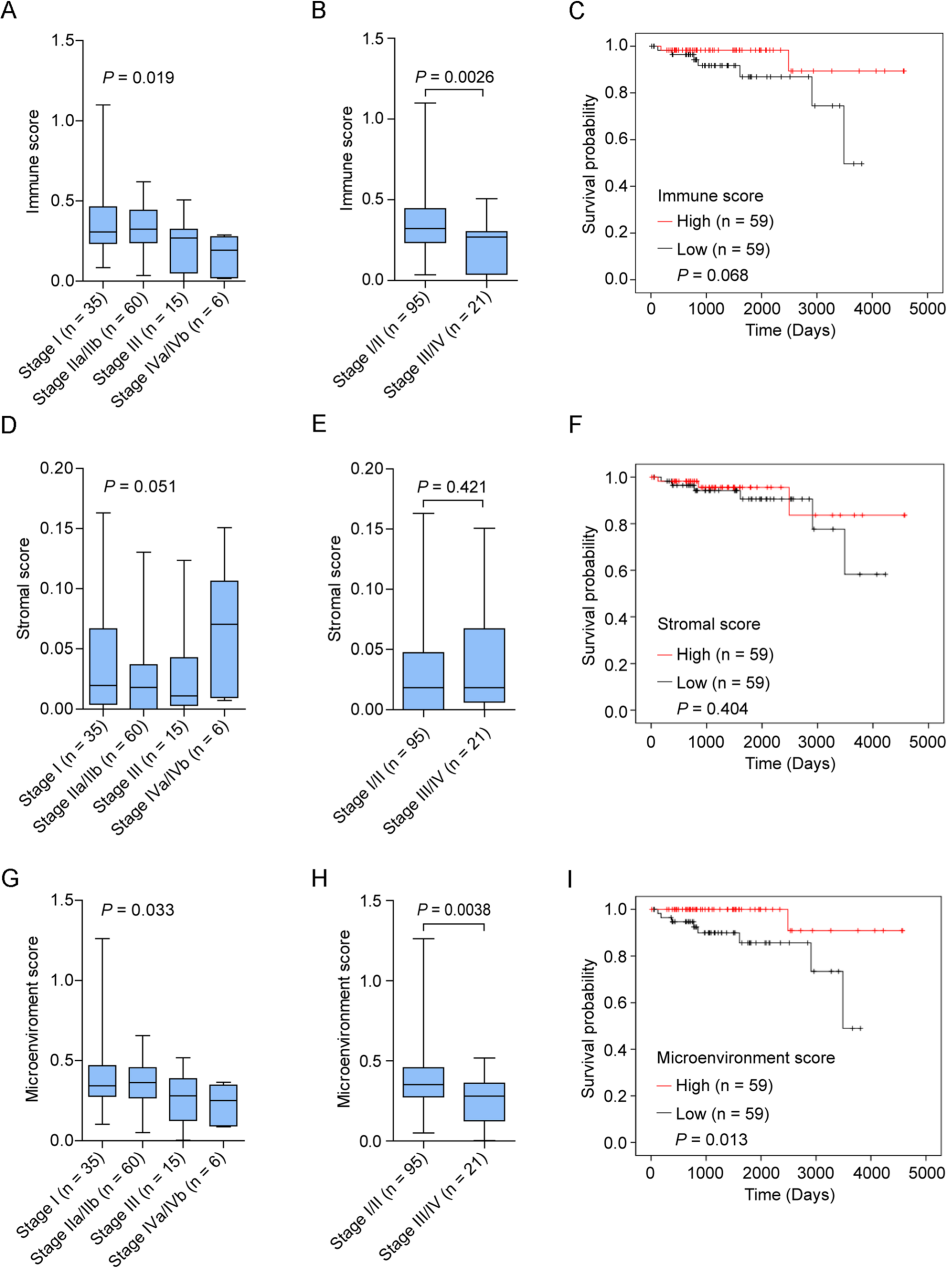
Associations between the TME and clinical features in TET patients. **A**, **B** Distribution of immune scores in the TCGA-TET cohort at different stages (**A**), early stage and late stage (**B**). **C** Kaplan–Meier curves of the overall survival of TCGA-TET cohort subgroups defined by the immune score. **D**, **E** Distribution of stromal scores in the TCGA-TET cohort at different stages (**D**), early stage and late stage (**E**). **F** Kaplan–Meier curves of the overall survival of TCGA-TET cohort subgroups defined by stromal score. **G**, **H** Distribution of TME scores in the TCGA-TET at different stages (**G**), early stage and late stage (**H**). **I** Kaplan–Meier curves of the overall survival of TCGA-TET cohort subgroups defined by the microenvironment score

We first explored the possibility of a relationship between TME components (i.e., immune and stromal components represented by immune and stromal scores, respectively) and clinical features in TETs. We found that the immune score decreased as the Masaoka stage increased (Fig. 1A, *P* = 0.019) and that the immune score was significantly lower in TETs at the late Masaoka stage (stage III/IV) than in those at the early Masaoka stage (stage I/II) (Fig. 1B, *P* = 0.0026). The relationship between the immune score and overall survival was further investigated, and patients with high immune scores tended to have a better prognosis than those with low immune scores (Fig. 1C, *P* = 0.068); however, the difference was not significant. Compared to those of stage I–III and early Masaoka stage TETs, the stromal scores tended to increase in stage IV (Fig. 1D) and late Masaoka stage TETs (Fig. 1E). However, there was no significant relationship between stromal score and overall survival in TET patients (Fig. 1F, *P* = 0.404). Similar to the findings obtained for the immune score, the microenvironment score (the sum of the immune and stromal scores) decreased as the Masaoka stage increased (Fig. 1G, *P* = 0.033), and the score was considerably lower in TETs at the late Masaoka stage than in those at the early Masaoka stage (Fig. 1H, *P* = 0.0038). Compared to patients with low microenvironment scores, those with high microenvironment scores had a better prognosis (Fig. 1I, *P* = 0.013). These findings show that TME components, especially immune components, play a clinical role in TETs.

### Identification of hub genes related to the immune response in TETs by WGCNA

**Fig. 2:**
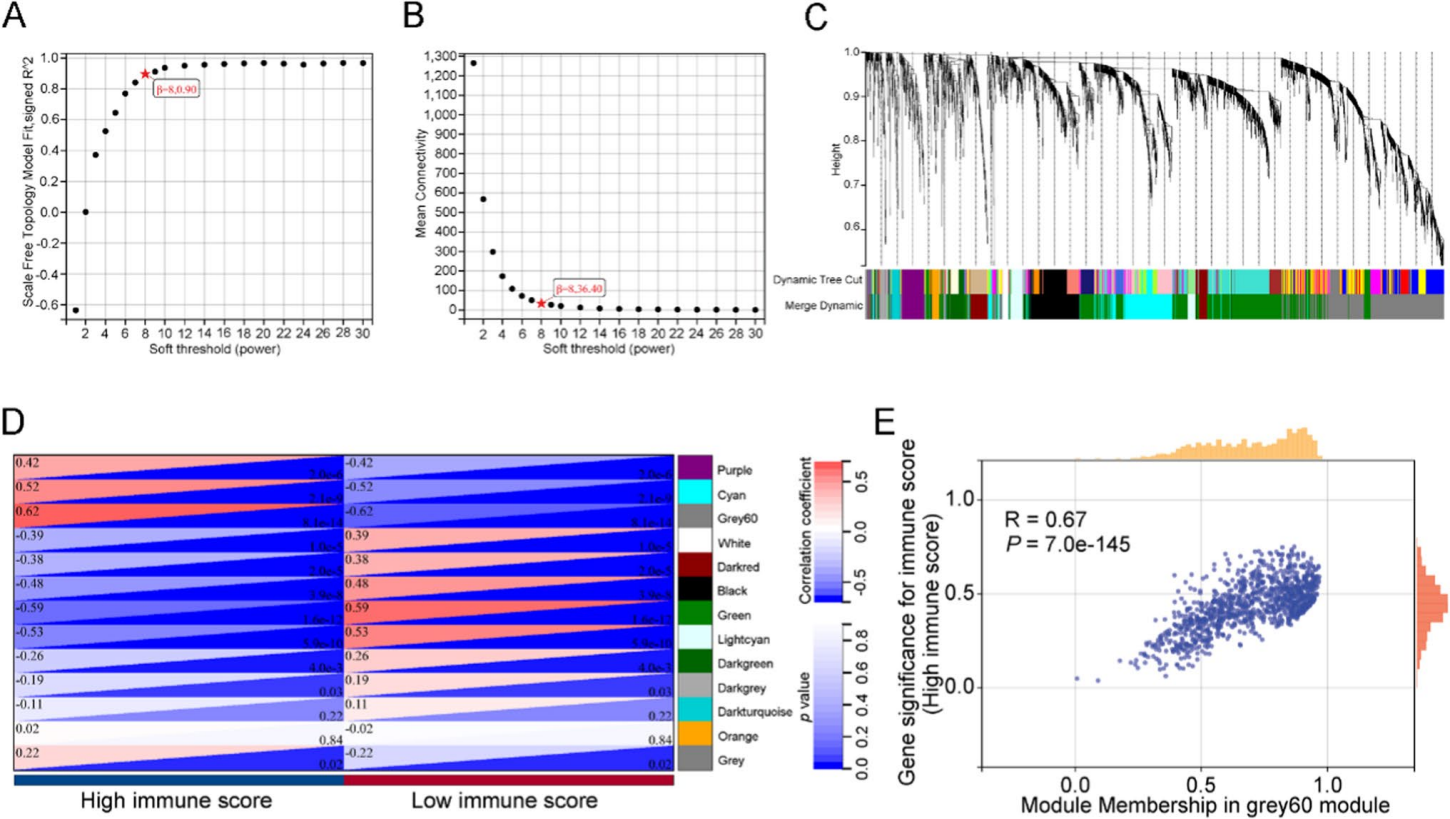
Identification of immune-related modules and key hub genes in TETs. **A** Analysis of the scale-free index for various soft-threshold powers (β). **B** Analysis of the mean connectivity for various soft-threshold powers. **C** Cluster dendrogram of the coexpression network modules (1-TOM). **D** Analysis of correlations between the modules and immune scores. **E** Scatter plots of MM and GS scores for genes in the grey60 module. *MM* module membership, *GS* gene significance

Next, we identified genes related to immune activity in TETs by determining the DEGs in TCGA TET samples with high versus low immune scores. A total of 1,455 upregulated and 2,406 downregulated genes were identified (Fig. S1 and Table S1). WGCNA was used to construct a network according to the DEG expression matrix. We set the soft threshold power to 8 to construct a scale-free network (Fig. 2A, B). DEGs with similar expression patterns were clustered into the same modules, yielding a total of 13 modules (Fig. 2C). The grey60 module had the strongest correlation with a high immune score (Fig. 2D, *R* = 0.62, *P* = 8.1e-14). Further analysis revealed that in the grey60 module, the MM and GS scores were strongly positively correlated (Fig. 2E, *R* = 0.67, *P* = 7.0e-145). Therefore, the grey60 module was analyzed for hub genes. With the criteria of MM > 0.9 and GS > 0.5, 84 hub genes were found in the grey60 module (Table S2). The hub genes were then assessed using univariate analysis, and almost all were related to overall survival in TET patients (Table S3). According to the *P* values, TCF3, HDAC7, OSBPL5, GATA3, RORC, PITPNM2, RHOH, LCK, TCF7, PTP4A2, CD1E, SH2D3C, TTC7A, MTA3, and ELOVL4 were among the top 15 hub genes related to overall survival in TET patients (Fig. S2, Fig. S3 and Table S3). Further single-cell analysis revealed that among the 15 hub genes, only RORC, PTP4A2, and MTA3 were abundant in thymic epithelial cells (Fig. S4).

### Deregulation of MTA3 is correlated with prognosis and the TME in TET patients

**Fig. 3:**
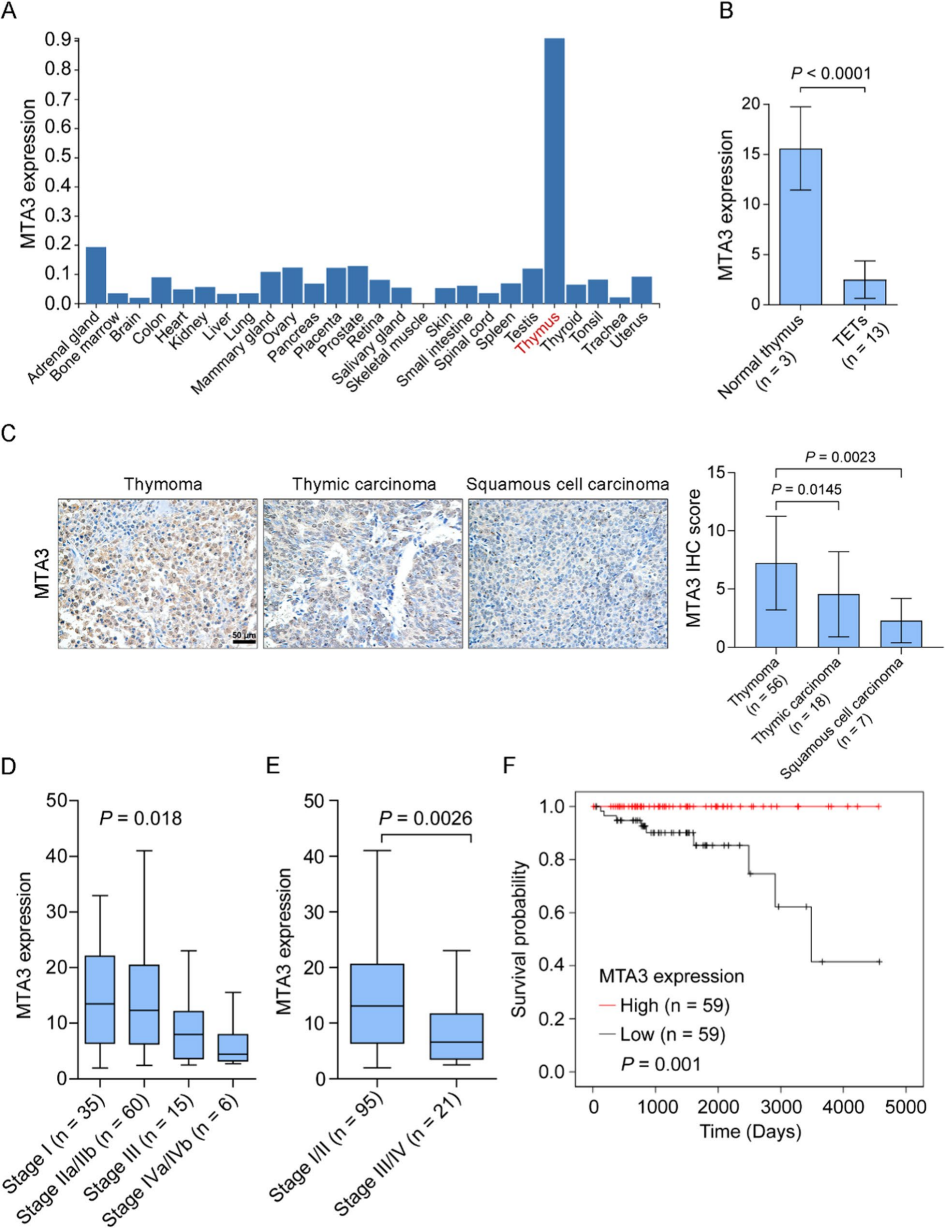
MTA3 is aberrantly expressed and clinically significant in TET patients. **A** The expression of MTA3 in various human tissues was investigated using the PaGenBase database. **B** The expression of MTA3 in TETs was investigated using the GEO dataset GSE79978. **C** IHC analysis of MTA3 in thymomas, thymic carcinomas, and squamous cell carcinomas. **D**, **E** Expression of MTA3 in the TCGA-TET cohort samples at different stages **(D**), early stage and late stage (**E**). **F** Kaplan–Meier curves of the overall survival of TCGA-TET cohort subgroups defined by MTA3 expression

MTA3 and RORC were further revealed to be notably expressed in thymus tissues (Fig. 3A and Fig. S5A); however, PTP4A2 was broadly expressed in various human tissues (Fig. S5B). Given that RORC has previously been associated with immune cell infiltration in TETs [39] and that MTA3 has previously been implicated in immune response regulation [19–22], we focused on the role of MTA3 in TETs. We found that MTA3 was significantly downregulated in thymic tumors (Fig. 3B and Fig. S6, *P* < 0.0001 for both). IHC revealed that the expression of MTA3 was downregulated in malignant thymic tumors, including thymic carcinomas and squamous cell carcinomas, compared to that in thymomas (Fig. 3C, *P* < 0.05 for both). Further analysis of the TCGA-TET cohort revealed that the expression level of MTA3 decreased as the Masaoka stage increased (Fig. 3D, *P* = 0.018) and was significantly lower in late Masaoka stage TETs than in early Masaoka stage TETs (Fig. 3E, *P* = 0.0026). We further investigated the correlation between MTA3 expression and various clinical characteristics in the TCGA-TET cohort. The results revealed that MTA3 was associated with Masaoka stage (Table 1, *P* = 0.01) and age (Table 1, *P* = 0.002) but not gender (Table 1, *P* = 0.712), myasthenia gravis (Table 1, *P* = 0.952), or radiation therapy (Table 1, *P* = 0.879). Furthermore, Kaplan‒Meier survival analysis revealed that TET patients with low MTA3 expression had poorer overall survival than those with high MTA3 expression (Fig. 3F, *P* = 0.001).

**Table 1:** Associations of MTA3 expression with clinicopathological variables in TET patients from TCGA

Variables	N	MTA3 expression		*P-*value
		Low, n (%)	High, n (%)	
All patients	118	59 (50.0)	59 (50.0)	
Age (Years)				
> 60	57	37 (64.9)	20 (35.1)	0.002
≤ 60	61	22 (36.1)	39 (63.9)	
Gender				
Male	62	32 (51.6)	30 (48.4)	0.712
Female	56	27 (48.2)	29 (51.8)	
Masaoka stage^*^				
I–II	95	43 (45.3)	52 (54.7)	0.01
III–IV	21	16 (76.2)	5 (23.8)	
Myasthenia gravis^*^				
No	81	40 (49.4)	41 (50.6)	0.952
Yes	34	17 (50.0)	17 (50.0)	
Radiation therapy*				
No	76	40 (52.6)	36 (47.4)	0.879
Yes	41	19 (46.3)	22 (56.7)	

*Clinical information of some patients is not complete

**Fig. 4:**
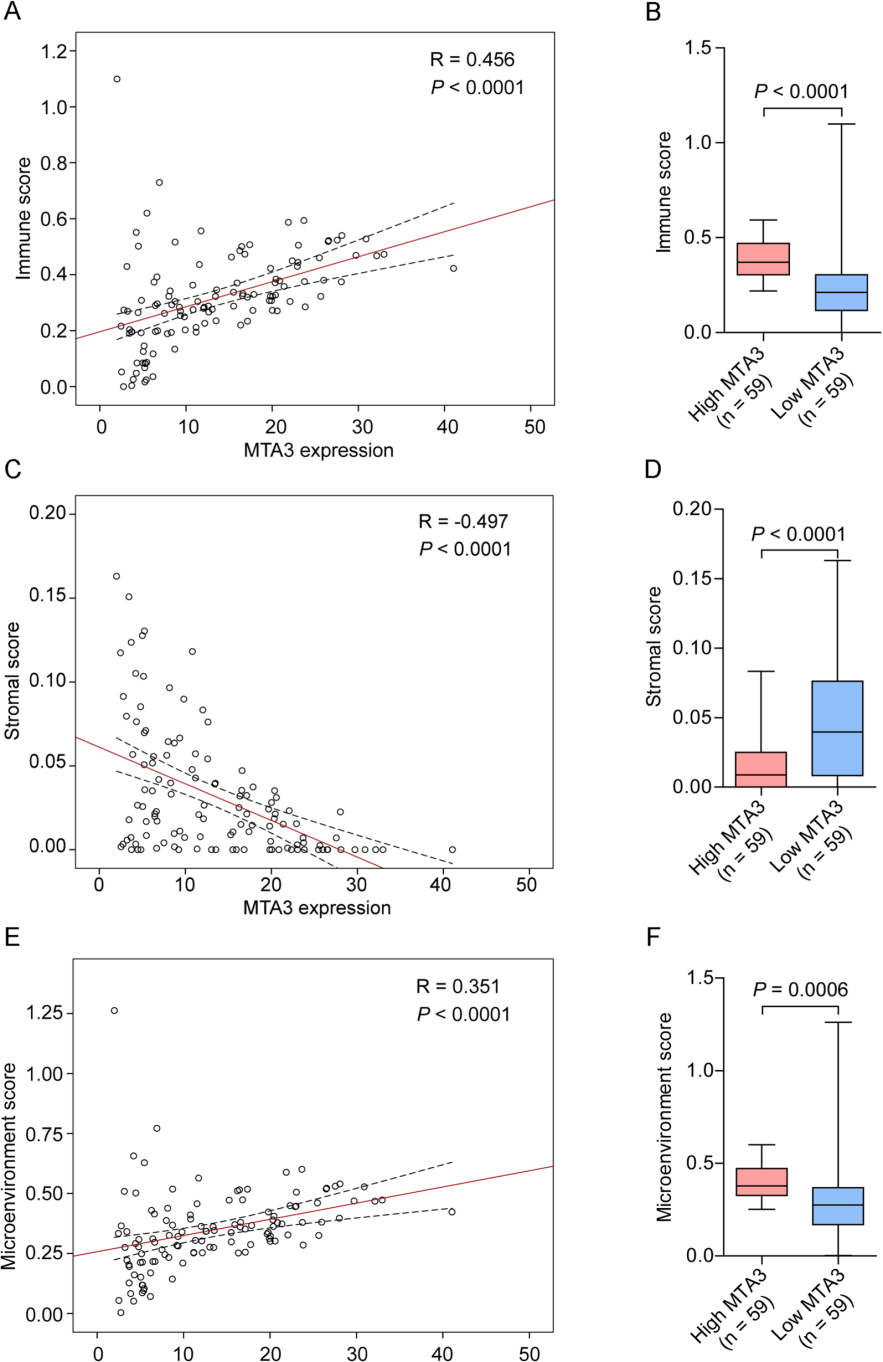
Deregulation of MTA3 is correlated with the TME in TETs. **A** Correlation analysis of MTA3 expression and immune score in the TCGA-TET cohort. **B** Distribution of immune scores in TCGA-TET cohort subgroups with high and low MTA3. **C** Correlation analysis of MTA3 expression and stromal score in the TCGA-TET cohort. **D** Distribution of stromal scores in the TCGA-TET cohort subgroups with high and low MTA3 expression. **E** Correlation analysis of MTA3 expression and the microenvironment score in the TCGA-TET cohort. **F** Distribution of tumor microenvironment scores in TCGA-TET cohort subgroups with high and low MTA3 expression

We further investigated the relationship between MTA3 expression and the TME and found that MTA3 expression was positively correlated with the immune score (Fig. 4A, *R* = 0.456, *P* < 0.0001). Moreover, TETs with high MTA3 expression had higher immune scores than those with low MTA3 expression (Fig. 4B, *P* < 0.0001). In contrast, MTA3 expression was inversely related to the stromal score (Fig. 4C, *R* = − 0.497, *P* < 0.0001), and TETs with high MTA3 expression had lower stromal scores than those with low MTA3 expression (Fig. 4D, *P* < 0.0001). Similar to the immune score, the MTA3 score was positively correlated with the microenvironment score (Fig. 4E, *R* = 0.351, *P* < 0.0001), and the microenvironment score was significantly greater in TETs with high MTA3 expression than in those with low MTA3 expression (Fig. 4F, *P* = 0.0006). Similar findings were obtained in an independent GEO TET cohort (GSE29695) (Fig. S7). Collectively, these findings suggest that the loss of MTA3 expression is associated with TET malignancy and that MTA3 may play a regulatory role in TME remodeling in TETs.

### MTA3 potentially predicts cytotoxic activity in TETs

**Fig. 5:**
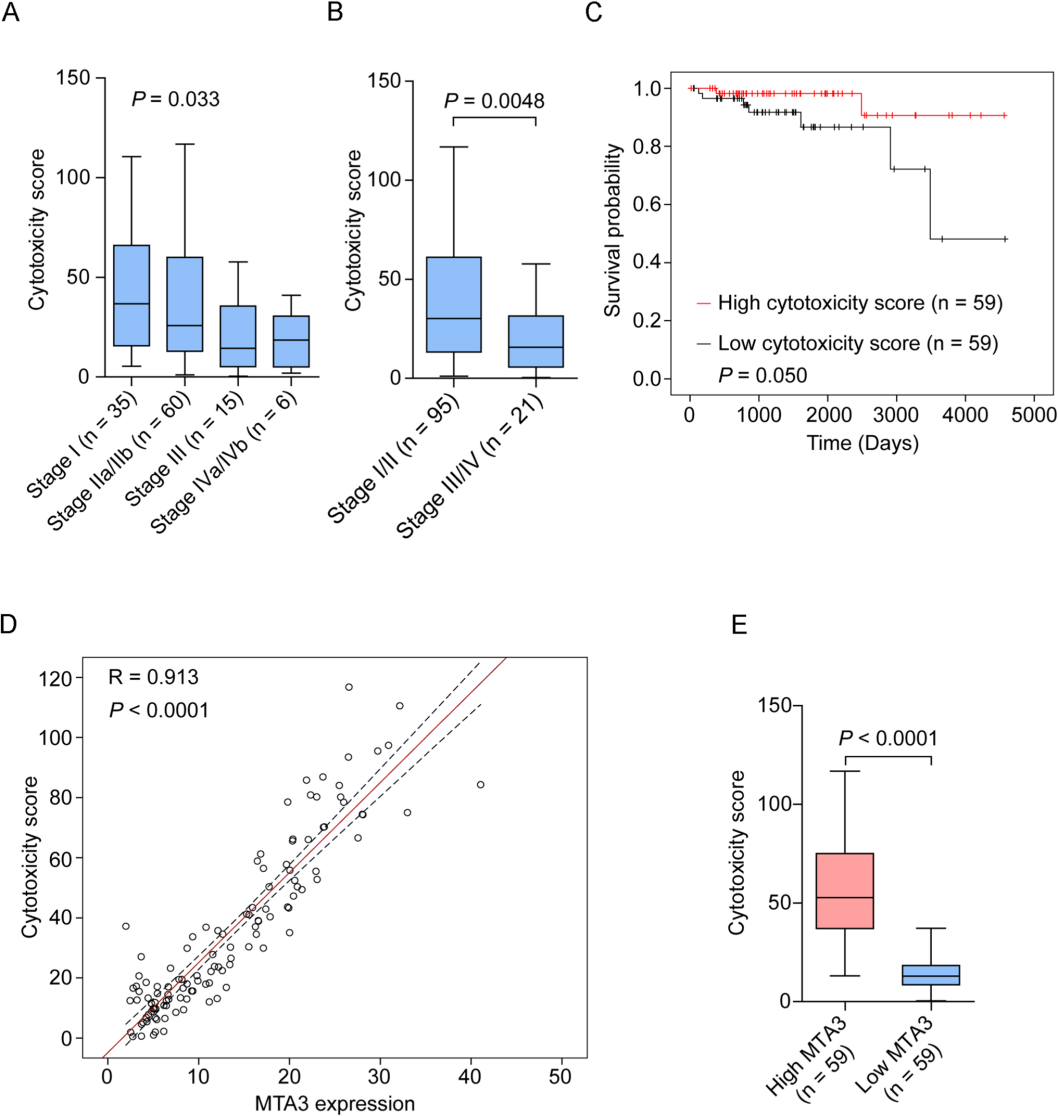
The cytotoxicity score is related to the clinical characteristics of TET patients and correlates with MTA3. **A**, **B** Distribution of cytotoxicity scores for TCGA-TETs at different stages (**A**), early-stage and late-stage (**B**). **C** Kaplan–Meier curves of the overall survival of TCGA-TET cohort subgroups defined by the cytotoxicity score. **D** Correlation analysis of MTA3 expression and cytotoxicity score in the TCGA-TET cohort. **E** Distribution of cytotoxicity scores in patients with high and low MTA3 expression in the TCGA-TET cohort

The discovery of a link between MTA3 and the TME prompted us to investigate the possible relationship between MTA3 and cytotoxic activity. We first found that the cytotoxicity score for TETs decreased as the Masaoka stage increased (Fig. 5A, *P* = 0.033), and the cytotoxicity scores for late Masaoka stage TETs were significantly lower than those for early Masaoka stage TETs (Fig. 5B, *P* = 0.0048). Moreover, an increased cytotoxicity score was associated with a better prognosis (Fig. 5C, *P* = 0.050). Furthermore, MTA3 expression strongly positively correlated with the cytotoxicity score (Fig. 5D, *R* = 0.913, *P* < 0.0001), and cytotoxicity scores were significantly greater for TETs with high MTA3 expression than for those with low MTA3 expression (Fig. 5E, *P* < 0.0001). These results suggest that the cytotoxicity score has a high prognostic predictive value and that MTA3 may predict cytotoxicity in TET patients.

### MTA3 expression correlates with immune cell infiltration in TETs

**Fig. 6:**
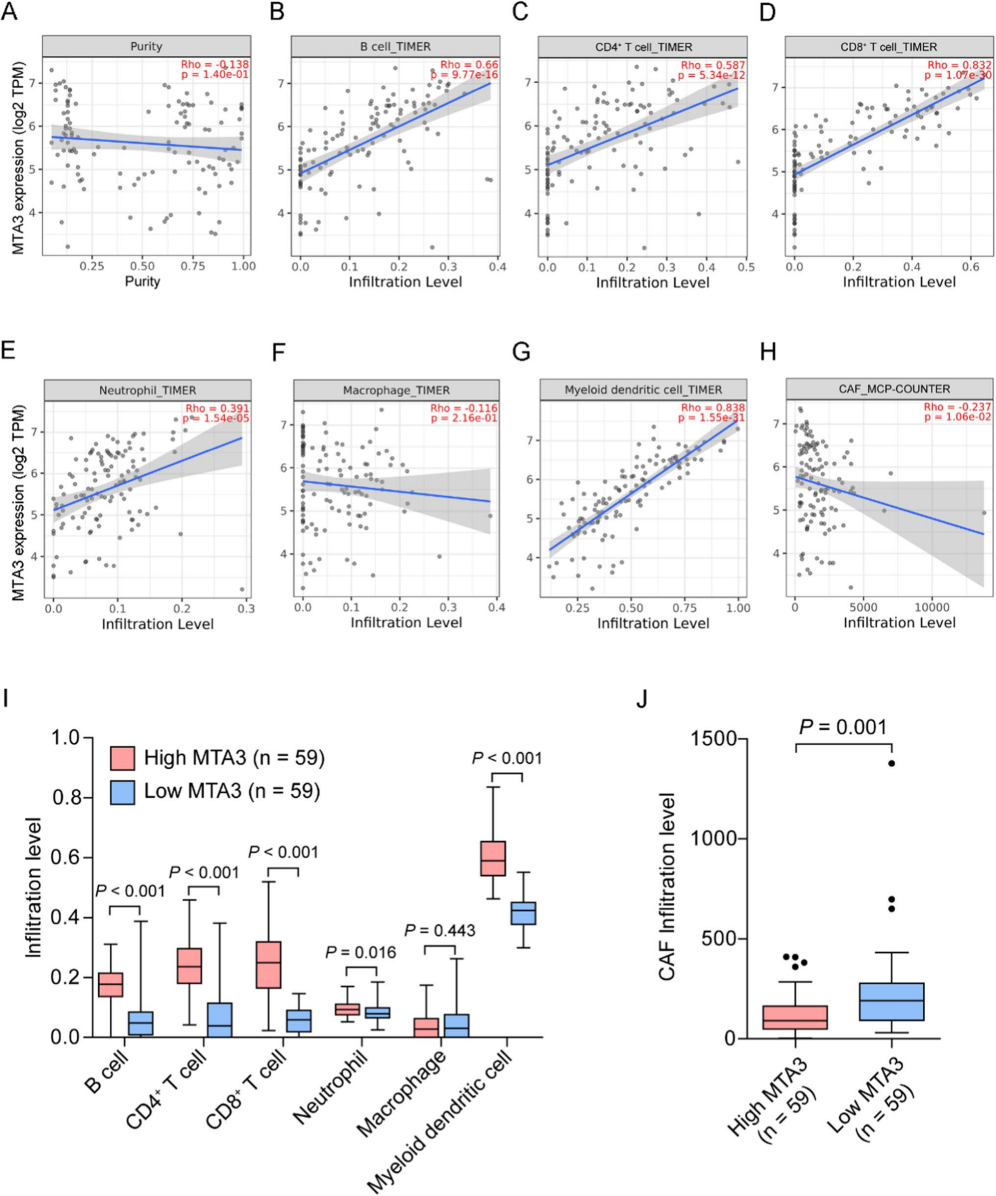
MTA3 expression is correlated with immune infiltration in TETs. **A**–**H** Correlations of MTA3 expression with tumor purity (**A**) and infiltration levels of B cells (**B**), CD4^+^ T cells (**C**), CD8^+^ T cells (**D**), neutrophils (E), macrophages (**F**), myeloid dendritic cells (**G**), and CAFs (**H**) in the TCGA-TET cohort. **I** Infiltration levels of B cells, CD4^+^ T cells, CD8^+^ T cells, neutrophils, macrophages, and myeloid dendritic cells in the TCGA-TET cohort subgroups with high and low MTA3 expression. **J** Infiltration level of CAFs in the TCGA-TET cohort subgroups with high and low MTA3 expression. TPM, transcripts per million

Given the above findings, we further investigated the potential relationship between MTA3 expression and immune cell infiltration in TETs using TIMER2.0. After adjustment for tumor purity (Fig. 6A), MTA3 expression positively correlated with the infiltration of B cells (Fig. 6B, *R* = 0.66, *P* = 9.77e-16), CD4^+^ T cells (Fig. 6C, *R* = 0.587, *P* = 5.34e-12), CD8^+^ T cells (Fig. 6D, *R* = 0.832, *P* = 1.07e-30), neutrophils (Fig. 6E, *R* = 0.391, *P* = 1.54e-05), and myeloid dendritic cells (Fig. 6G, *R* = 0.838, *P* = 1.55e-31). However, there was no significant correlation between MTA3 expression and macrophage infiltration (Fig. 6F, *R* = − 0.116, *P* = 2.16e-01). Moreover, MTA3 was inversely correlated with CAF infiltration (Fig. 6H, *R* = − 0.237, *P* = 1.06e-02). We subsequently compared the infiltration levels of each type of immune cell between the MTA3-high and MTA3-low TETs. B cell, CD4^+^ T cell, CD8^+^ T cell, neutrophil, and myeloid dendritic cell infiltration levels were significantly greater in MTA3-high TETs than in MTA3-low TETs (Fig. 6I, *P* < 0.05 for all); however, there was no significant difference in macrophage infiltration (Fig. 6I, *P* = 0.443). In addition, TETs with high MTA3 expression had a lower CAF infiltration level than those with low MTA3 expression (Fig. 6J, *P* = 0.001). Similar results were found in an independent GEO TET cohort (GSE29695) (Fig. S8).

To better understand the role of MTA3 in immune cell infiltration, we analyzed the correlations between MTA3 and the expression of various immune cell markers in TETs using the TIMER database. As shown in Table 2, MTA3 expression correlated with most immune cell markers. Notably, after adjustment for tumor purity, MTA3 expression remained strongly correlated with markers of CD8^+^ T cells, including CD8A (Table 2, R = 0.898, *P* = 3.87e-42) and CD8B (Table 2, R = 0.846, *P* = 1.10e-32), as well as general T-cell markers, including CD3D (Table 2, R = 0.678, *P* = 8.70e-17), CD3E (Table 2, R = 0.862, *P* = 4.54e-35), and CD2 (Table 2, R = 0.815, *P* = 1.74e-28). On the other hand, MTA3 expression was negatively correlated with multiple markers of T-cell exhaustion, including CTLA4 (Table 2, R = − 0.481, *P* = 5.36e-08), LAG3 (Table 2, R = − 0.611, *P* = 4.15e-13), and TIM-3 (Table 2, R = − 0.365, *P* = 5.92e-05). These data suggest that MTA3 may play a role in TME modulation by regulating immune cell infiltration.

**Table 2:** Correlation between MTA3 and gene markers of immune cells in TETs from TCGA

Description	Gene markers	None		Purity	
		Cor	*P-*value	Cor	*P-*value
CD8^+^ T cell	CD8A	0.898	0e + 00	0.898	3.87e-42
	CD8B	0.852	0e + 00	0.846	1.10e-32
T cell (general)	CD3D	0.695	0e + 00	0.678	8.70e-17
	CD3E	0.863	0e + 00	0.862	4.54e-35
	CD2	0.817	0e + 00	0.815	1.74e-28
B cell	CD19	− 0.174	5.7e-02	− 0.216	2.06e-02
	CD79A	0.544	1e-10	0.517	3.30e-09
Monocyte	CD86 (B7-2)	− 0.414	3.29e-06	− 0.402	8.45e-06
	CD115 (CSF1R)	− 0.422	2.04e-06	− 0.397	1.11e-05
TAM	CCL2	− 0.214	1.91e-02	− 0.2	3.17e-02
	CD68	− 0.266	3.39e-03	− 0.25	7.09e-03
	IL10	− 0.06	5.15e-01	− 0.059	5.29e-01
M1 Macrophage	INOS (NOS2)	− 0.214	1.87e-02	− 0.18	5.40e-02
	IRF5	− 0.442	5.76e-07	− 0.447	5.50e-07
	COX2 (PTGS2)	− 0.399	7.81e-06	− 0.381	2.72e-05
M2 Macrophage	CD163	− 0.246	6.83e-03	− 0.239	9.99e-03
	VSIG4	− 0.296	1.09e-03	− 0.269	3.64e-03
	MS4A4A	− 0.017	8.57e-01	− 0.005	9.57e-01
Neutrophils	CD66B (CEACAM8)	0.29	1.3e-03	0.291	1.61e-03
	CD11B (ITGAM)	− 0.137	1.36e-01	− 0.117	2.15e-01
	CCR7	0.187	4.14e-02	0.155	9.79e-02
Natural kill cell	KIR2DL1	− 0.214	1.92e-02	− 0.193	3.89e-02
	KIR2DL3	− 0.172	6.06e-02	− 0.152	1.05e-01
	KIR2DL4	− 0.435	6.68e-07	− 0.407	6.33e-06
	KIR3DL1	− 0.303	7.68e-04	− 0.269	3.59e-03
	KIR3DL2	0.055	5.51e-01	0.078	4.09e-01
	KIR3DL3	0.052	5.7e-01	0.078	4.07e-01
	KIR2DS4	− 0.051	5.77e-01	− 0.017	8.61e-01
Dendritic cell	HLA-DPB1	− 0.339	1.7e-04	− 0.341	1.96e-04
	HLA-DQB1	− 0.204	2.58e-02	− 0.25	7.14e-03
	HLA-DRA	− 0.342	1.44e-04	− 0.341	1.90e-04
	HLA-DPA1	− 0.281	1.98e-03	− 0.265	4.24e-03
	BDCA1 (CD1C)	0.896	0e + 00	0.896	1.23e-41
	BDCA4 (NRP1)	− 0.157	8.75e-02	− 0.115	2.20e-01
	CD11C (ITGAX)	− 0.341	1.54e-04	− 0.332	2.93e-04
Th1	T-BET (TBX21)	− 0.217	1.75e-02	− 0.214	2.18e-02
	STAT4	− 0.079	3.93e-01	− 0.055	5.62e-01
	STAT1	− 0.302	8.58e-04	− 0.3	1.14e-03
	IFN-γ (IFNG)	− 0.516	1.58e-09	− 0.516	3.74e-09
	TNF-α (TNF)	− 0.404	5.78e-06	− 0.429	1.73e-06
Th2	GATA3	0.886	0e + 00	0.885	2.18e-39
	STAT6	− 0.08	3.85e-01	− 0.035	7.10e-01
	STAT5A	− 0.279	2.13e-03	− 0.232	1.25e-02
	IL13	− 0.165	7.12e-02	− 0.185	4.77e-02
Th17	STAT3	− 0.241	8.17e-03	− 0.203	2.94e-02
	IL17A	− 0.21	2.61e-02	− 0.209	2.48e-02
Tfh	BCL6	0.359	6.44e-05	0.377	3.25e-05
	IL21	− 0.113	2.17e-01	− 0.12	2.00e-01
Treg	FOXP3	− 0.32	3.9e-04	− 0.324	4.20e-04
	CCR8	0.473	5e-08	0.455	3.32e-07
	STAT5B	0.302	8.52e-04	0.335	2.50e-04
	TGFβ (TGFB1)	− 0.083	3.67e-01	− 0.066	4.82e-01
	IL2RA (CD25)	− 0.296	1.06e-03	− 0.279	2.55e-03
T cell exhaustion	PD-1 (PDCD1)	0.662	0e + 00	0.651	3.50e-15
	CTLA4	− 0.461	1.65e-07	− 0.481	5.36e-08
	LAG3	− 0.614	0e + 00	− 0.611	4.15e-13
	TIM-3 (HAVCR2)	− 0.357	6.94e-05	− 0.365	5.92e-05

Cor, R-value of Spearman’s correlation; None, correlation without adjustment; Purity, correlation adjusted by purity. *TAM* tumor-associated macrophage, *Th* T helper cell, *Tfh* follicular helper T cell, *Treg* regulatory T cell

### MTA3 is related to the activation of immune pathways in TETs

**Fig. 7:**
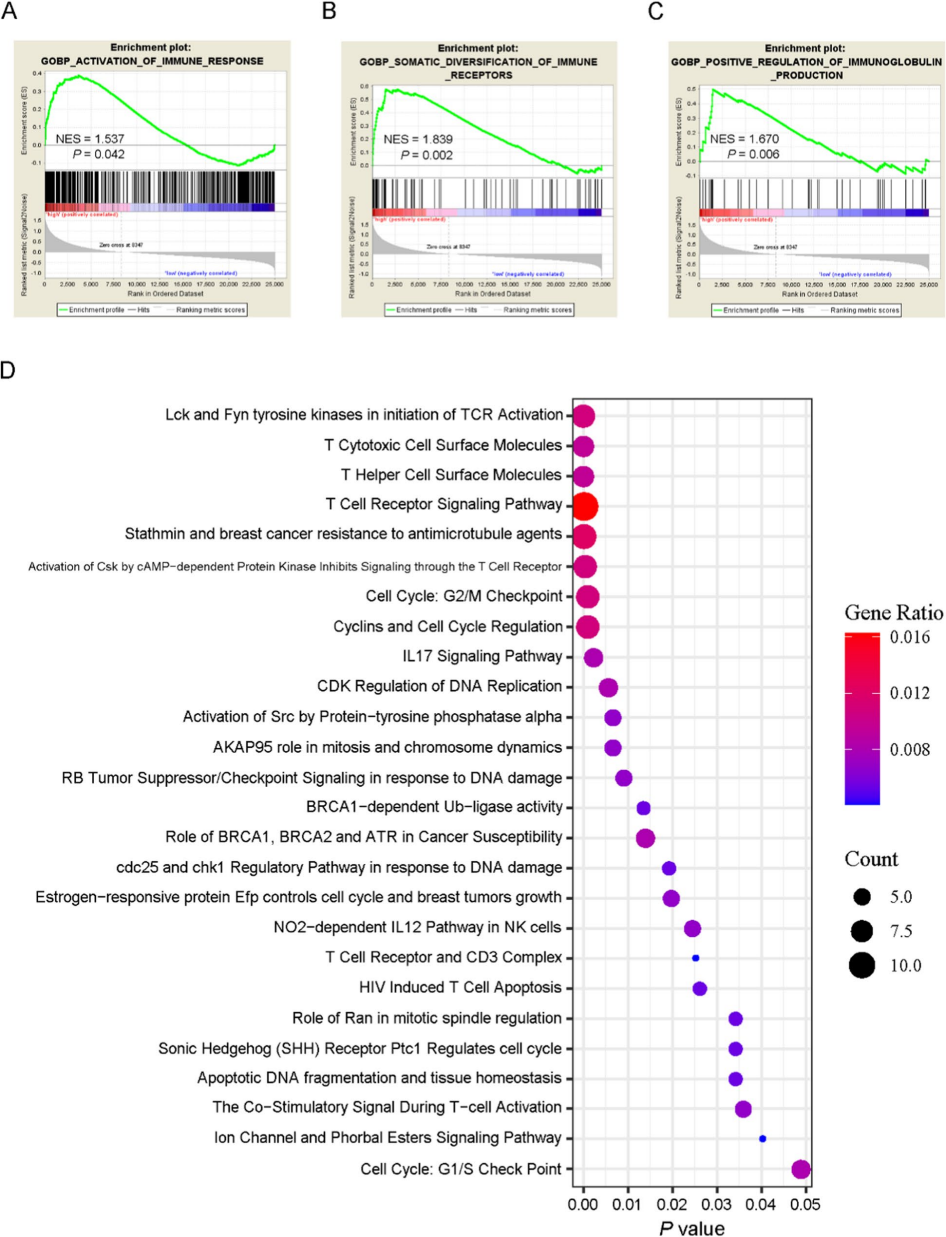
MTA3 may be involved in activating immune pathways in TETs. **A**–**C** GSEA plots of the enrichment of the “GOBP_ACTIVATION_OF_IMMUNE_RESPONSE” signature (**A**), the “GOBP_SOMATIC_DIVERSIFICATION_OF_IMMUNE_RECEPTORS” signature (**B**), and the “GOBP_POSITIVE_REGULATION_IMMUNOGLOBULIN_PRODUCTION” signature (**C**) in MTA3^high^ versus MTA3^low^ TCGA-TET subgroups.** D** BIOCARTA pathway enrichment analysis of the genes positively correlated with MTA3

To further investigate the possible mechanisms underlying the role of MTA3 in immune cell infiltration, we applied GSEA and pathway enrichment analysis to identify the signaling pathways that might be affected by MTA3. As revealed by GSEA, the signatures for immune response were found to be enriched in patients with high MTA3, including GOBP_ACTIVATION_OF_IMMUNE_RESPONSE (Fig. 7A, normalized enrichment score (NES) = 1.537, *P* = 0.042), GOBP_SOMATIC_DIVERSIFICATION_OF_IMMUNE_RECEPTORS (Fig. 7B, NES = 1.839, *P* = 0.002), and GOBP_POSITIVE_REGULATION_OF_IMMUNOGLOBULIN_PRODUCTION (Fig. 7C, NES = 1.670, *P* = 0.006). We further explored the genes that positively correlated with MTA3 in the TCGA-TET cohort using the UALCAN database, and the genes strongly positively correlating with MTA3 expression (Table S4, R ≥ 0.7) were assessed by BIOCARTA pathway enrichment analysis. The results revealed that these genes are enriched in various immune-related molecules and pathways, including “T cytotoxic cell surface molecules”, “T helper cell surface molecules”, “T cell receptor signaling pathway”, and “IL17 signaling pathway” (Fig. 7D). These findings suggest that MTA3 may regulate the TME by modulating immune-related signaling.

## Discussion

In this study, we found that the TME has clinical significance in TETs, and MTA3 was identified as a key gene associated with immune score. Moreover, the expression of MTA3 was deregulated in TETs. Regarding clinical features, MTA3 expression negatively correlated with the Masaoka stage, suggesting that MTA3 may act as a tumor suppressor in TETs, inhibiting cancer progression. Furthermore, MTA3 was identified as a prognostic marker in TET patients and correlated with immune cell infiltration. In addition, MTA3 is likely involved in numerous immune pathways in TETs.

MTA3 suppresses various malignant behaviors (e.g., invasion, metastasis, and stemness) in many cancers [9–15], and its downregulation predicts poor patient prognosis in various cancers [9, 14–17]. Similarly, we found that MTA3 was downregulated in thymic tumors and that decreased MTA3 was associated with shorter overall survival in TET patients. Notably, MTA3 expression was lower in other TET subtypes than in thymomas, suggesting that MTA3 could be a biomarker for distinguishing thymomas from other TET subtypes. As expected, IHC experiments revealed that MTA3 was expressed primarily in the nucleus, suggesting that it plays a transcriptional regulatory role in TETs, consistent with its role in transcriptional regulation [8]. In addition, MTA3 tended to be inversely correlated with the EMT signature “HALLMARK_EPITHELIAL_MESENCHYMAL_TRANSITION” in TETs (Fig. S9, NES = − 1.370, *P* = 0.158), suggesting that MTA3 may play a role in the regulation of aggressiveness of TETs, consistent with its role in EMT regulation [9, 18].

Our findings suggest that the TME, especially its immune components, is associated with TET progression and patient prognosis. In support of these findings, numerous studies have demonstrated the importance of the immune microenvironment in cancer development [40], and the TME composition varies among the pathogenic subtypes of TETs [41]. Previous studies have shown that immune cells are important components of the TME in TETs and are associated with patient prognosis [42, 43], highlighting the importance of studying the interaction between tumor cells and immune cells.

Recently, programmed cell death 1 (PD-1)/programmed cell death 1 ligand 1 (PD-L1) inhibitors have been evaluated in phase I/II studies for treating TETs and have shown promising antitumor activity [6, 7]. However, immune checkpoint inhibitors clearly benefit only a subset of TET patients [6, 7]. Because the TME plays a critical role in patients’ response to immune checkpoint inhibitors [44], identifying the molecules involved in TME regulation is critical. Here, we found that MTA3 was closely related to both the immune and stromal components of the TME. Moreover, MTA3 was strongly associated with cytotoxicity scores and strongly positively correlated with infiltrating CD8^+^ T cells. A greater CD8^+^ T-cell fraction was observed in the MTA3-high patient group, suggesting that MTA3 may play a role in CD8^+^ T-cell activation or recruitment. One recent study demonstrated that CD8^high^ tumor-infiltrating lymphocytes are associated with better outcomes in advanced thymic carcinoma patients [43]. In the present study, MTA3 expression was also found to be positively correlated with the infiltration of other immune cells, including B cells, CD4^+^ T cells, neutrophils, and myeloid dendritic cells, while it was negatively correlated with the infiltration of CAFs. There is considerable evidence that CAFs can aid immune evasion by recruiting immunosuppressive cells into the tumor stroma [45]. Given the above findings, MTA3 may exert antitumor effects by improving the immune response and suppressing immune escape. Notably, MTA3 was inversely correlated with most T-cell exhaustion markers (i.e., CTLA4, LAG3, and TIM-3) but strongly positively associated with PD-1 (Table 2, R = 0.651, *P* = 3.50e-15), and patients with high MTA3 levels had higher PD-1 levels (Fig. S10, *P* < 0.0001). PD-1 is highly expressed in tumor-infiltrating lymphocytes in TETs [46, 47]. These findings suggest that TET patients with high MTA3 expression may benefit from PD-1-targeted therapy. Because the thymus is where the adaptive immune response develops and T cells mature, immune checkpoint inhibitors may cause severe autoimmune toxicity when administered to TET patients [48, 49], thus limiting their clinical utility. Therefore, there is an urgent need to identify reliable biomarkers to help select patients who may benefit from this treatment, optimize its efficacy, and limit autoimmune toxicity. Based on the findings that TET patients with high MTA3 expression had high PD-1 expression and abundant CD8^+^ T cells, suggesting that MTA3 may be a potential biomarker for accurately identifying a group of TET patients who may benefit from immune checkpoint inhibitor therapies, thereby optimizing their efficacy, and limiting autoimmune toxicity. It is worthwhile to investigate the role of MTA3 in autoimmune toxicity.

Aside from the canonical role of MTA3 in cancer, several lines of evidence point to a role for MTA3 in immune regulation. As a transcriptional corepressor of BCL6, MTA3 regulates B-cell development [19–21] and modulates CD4 T cell fate and function via repression of PR/SET domain 1 (PRDM1) [22]. Consistent with these findings, MTA3 was favorably associated with B-cell infiltration and inversely correlated with PRDM1 expression (Fig. S11, R = − 0.319, *P* = 0.0004). Recently, MTA3 was found to play a key role in immune tolerance in melanoma by upregulating PD-L1 [50]. In contrast, we found a negative correlation between MTA3 and PD-L1 in TETs (Fig. S12, R = − 0.273, *P* = 0.003), suggesting that MTA3 plays a complex role in cancer immunity in different cancer types. In addition to MTA3, MTA1 and MTA2 have been shown to play important roles in T cell function [51, 52]. Intriguingly, we found that MTA3 was associated with the activation of the immune response and immune pathways in TETs. Whether MTA3 plays a role in the development and function of immune cells in cancer, especially in TETs, is worthy of further investigation.

Nonetheless, there are some limitations to this study. The clinical importance of MTA3 expression and its association with immune cell infiltration in TETs were mainly derived from TCGA and GEO patient cohorts; thus, these findings should be validated in additional independent cohorts. Further study is needed to determine whether MTA3 is associated with immune cell infiltration and the efficacy of immunotherapy in actual TET patients. Furthermore, in vivo and in vitro experiments are required to determine MTA3’s role in TETs and investigate the underlying mechanisms.

## Conclusion

In this study, it was found that MTA3 has clinical implications for TETs regarding prognosis and immune status. MTA3 deficiency is linked to a poor clinical outcome, and MTA3 might be involved in shaping the TME in TETs. Thus, the present study’s findings suggested that MTA3 has the potential to be a novel prognostic and immunological biomarker for TET patients.

### Supplementary Information

Supplementary Material 1.

Supplementary Material 2.

Supplementary Material 3.

Supplementary Material 4.

Supplementary Material 5.

## Data Availability

All data related to this study are included in the article/supplementary materials.
