# Supplementary material for: Prognostic and immunological significance of metastasis-associated protein 3 in patients with thymic epithelial tumors
Source: Discov Oncol. 2024 Jun 9;15:216. doi: 10.1007/s12672-024-01066-1 (PMC11162987; doi:10.1007/s12672-024-01066-1)
Supplement: Supplementary file 1 — Supplementary Material 1. [file 12672_2024_1066_MOESM1_ESM.docx]

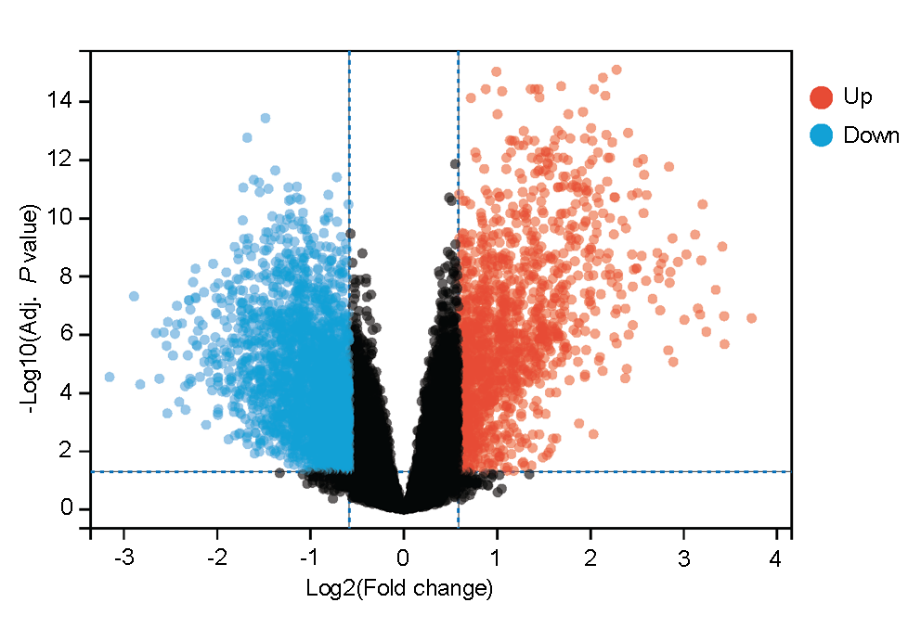


**Fig. S1.** Identification of the DEGs that may be related to immune activity in TETs. The DEGs in the immune score^High^ versus immune score^Low^ TCGA-TETs were shown in volcano plots. DEGs, differentially expressed genes.


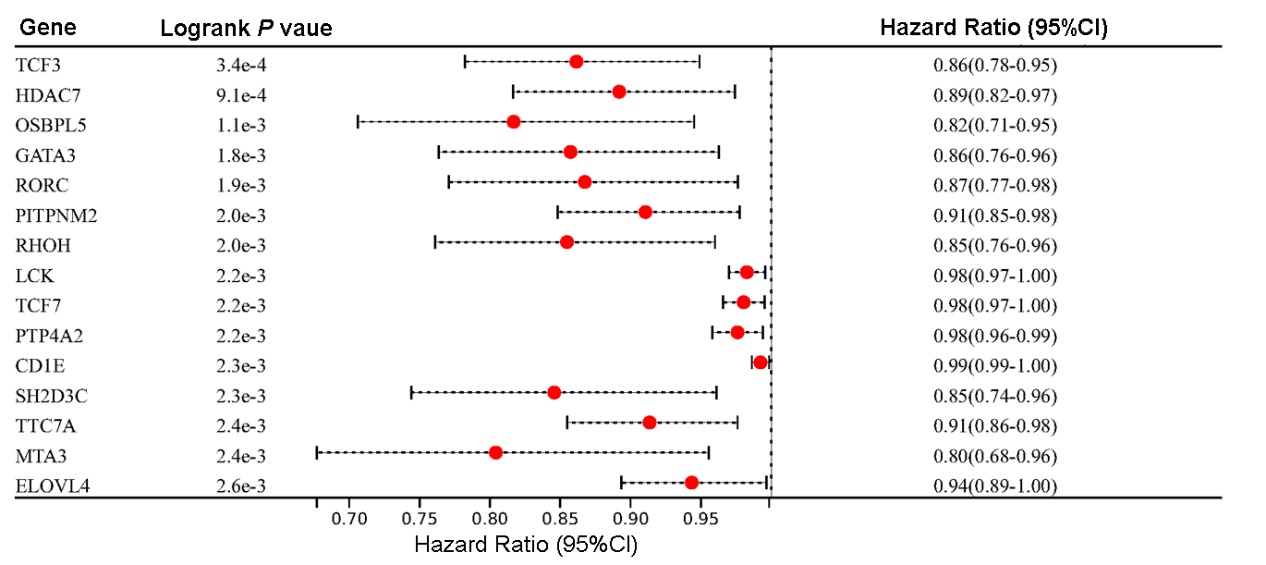


**Fig. S2.** The association of hub genes and prognosis in TET patients. The top 15 hub genes that were associated with the overall survival of TCGA TET patients as revealed by univariate analysis.


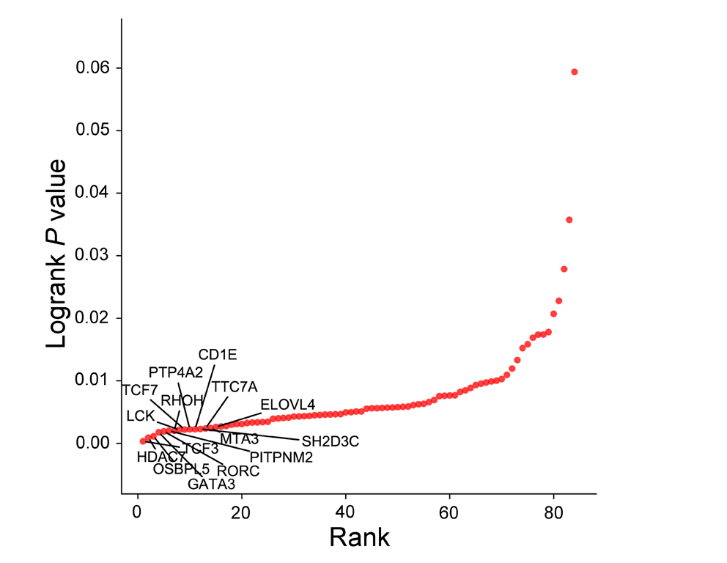


**Fig. S3.** Rank of hub genes' relation with overall survival in TET patients. The association of hub genes and overall survival in TCGA TET patients was ranked by logrank *P* value.


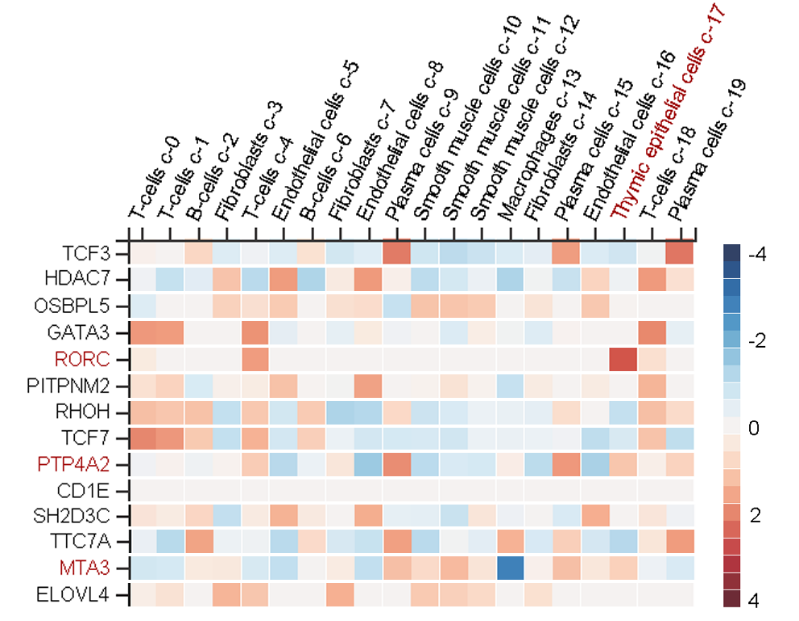


**Fig. S4.** Expression of top 15 hub genes in different single cell type clusters in the thymus. Single-cell analysis of top 15 hub genes in different single-cell type clusters in the thymus using the Human Protein Atlas database.


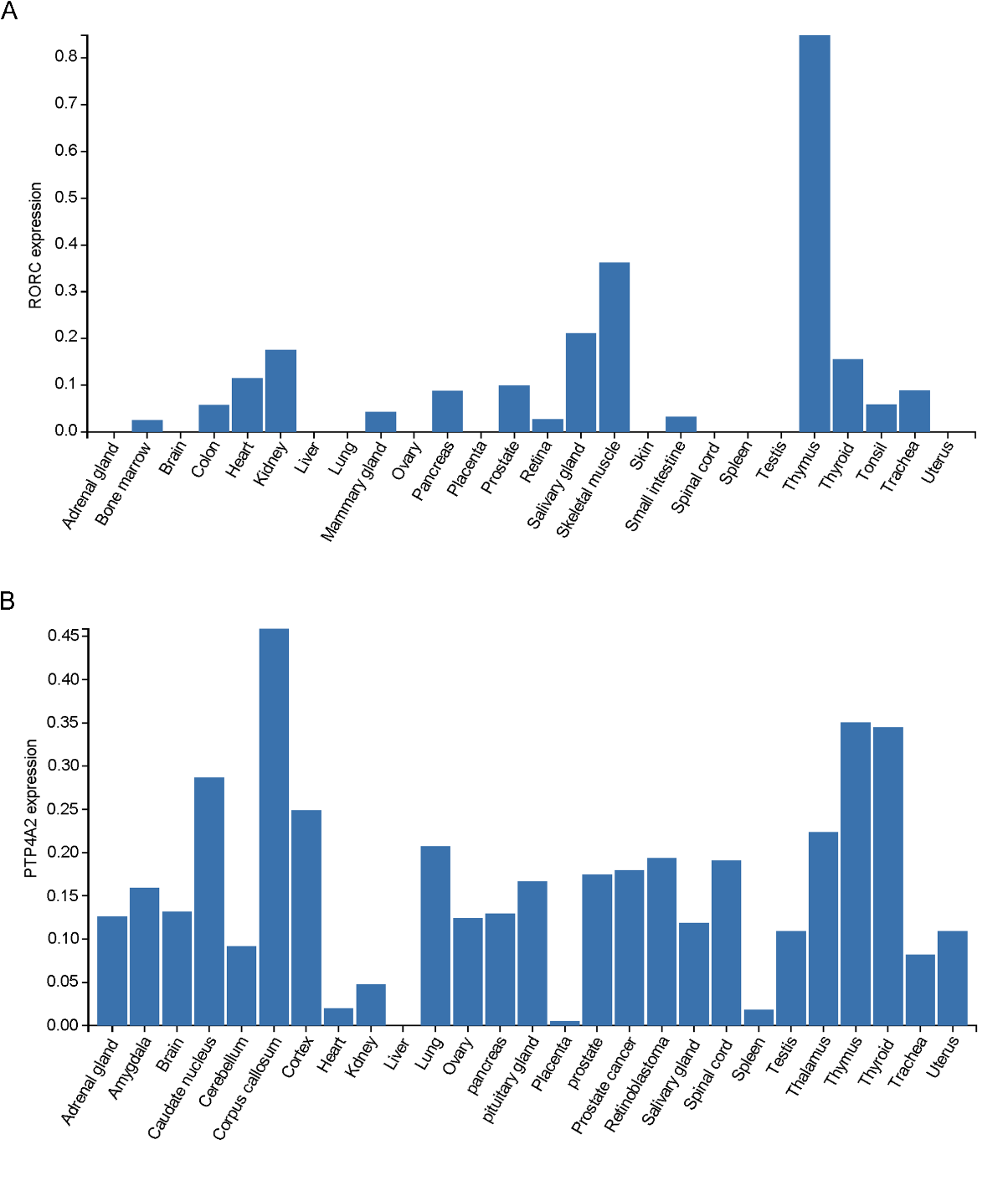


**Fig. S5.** Expression of RORC and PTP4A2 in various human tissues. (**A**, **B**) The expression of RORC (**A**) and PTP4A2 (**B**) across human tissues was investigated in the PaGenBase database.


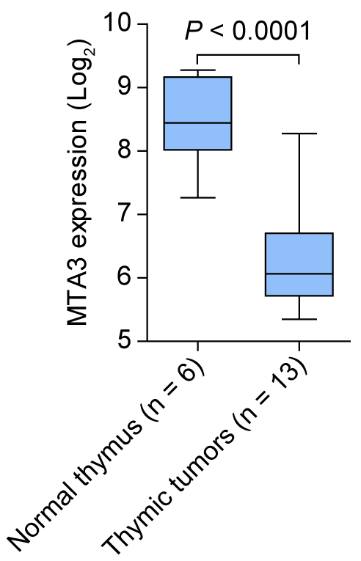


**Fig. S6.** Downregulation of MTA3 in thymic tumors. The expression of MTA3 in thymic tumors (including 11 thymic carcinoids and 2 thymomas) was investigated in the GEO dataset GSE177522.


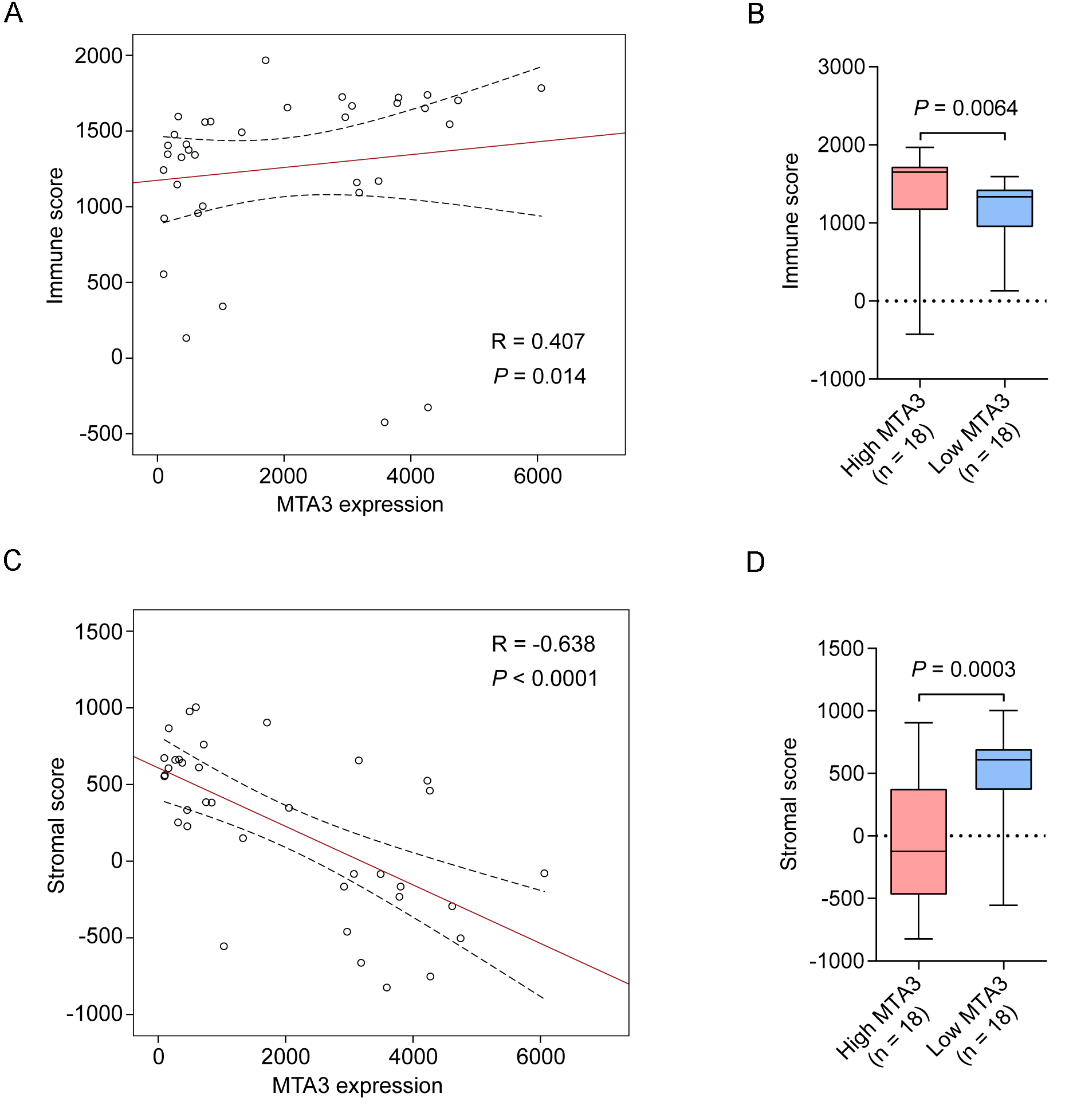


**Fig. S7.** MTA3 is correlated with the TME in TETs. (**A**) Correlation analysis of MTA3 expression and immune score in the GEO TET cohort (GSE29695). (**B**) Distribution of immune scores in the GEO TET cohort (GSE29695) subgroups with high and low MTA3 expression. (**C**) Correlation analysis of MTA3 expression and stromal score in the GEO TET cohort (GSE29695). (**D**) Distribution of stromal scores in the GEO TET cohort (GSE29695) subgroups with high and low MTA3 expression.

**
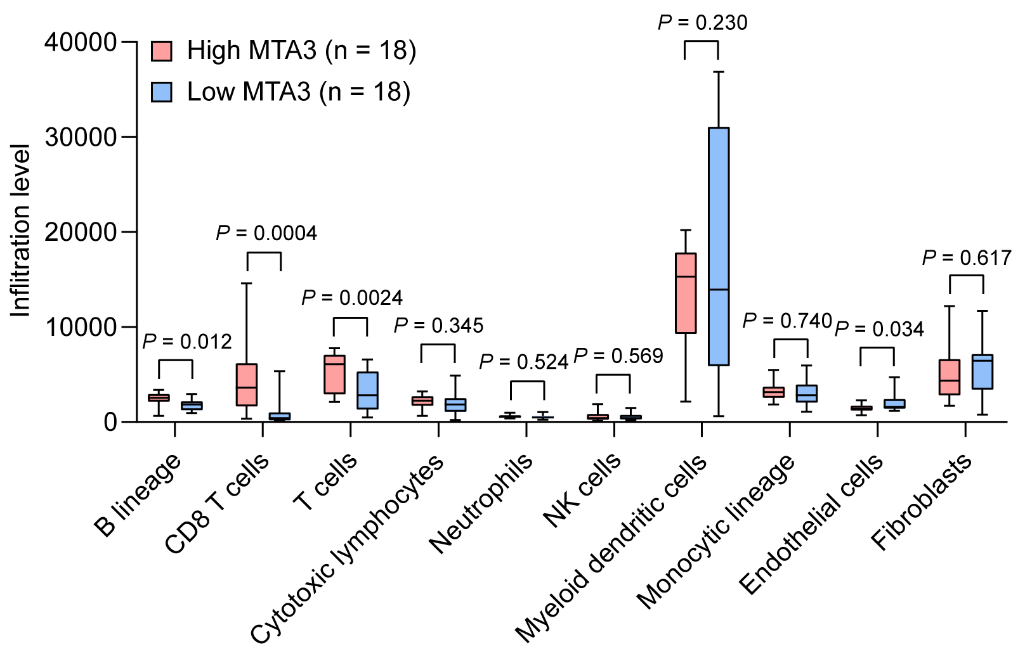
**

**Fig. S8.** MTA3 expression is correlated with immune infiltration in TETs. Infiltration levels of the indicated cells in the GEO TET cohort (GSE29695) subgroups with high and low MTA3 expression.


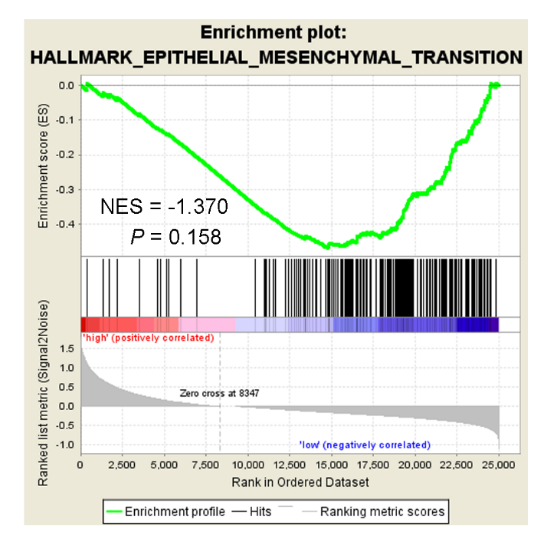


**Fig. S9.** MTA3 has a negative relationship with EMT signature in TETs. GSEA plots of enrichment of "HALLMARK_EPITHELIAL_MESENCHYMAL_TRANSITION" signature in MTA3^high^ versus MTA3^low^ TCGA-TETs.


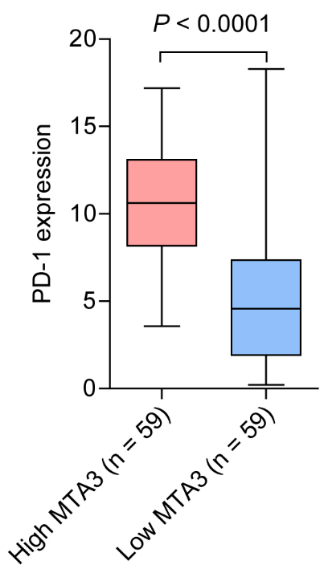


**Fig. S10.** PD-1 is highly expressed in TETs with high MTA3 expression. Expression of PD-1 in TCGA-TET cohort subgroups with high and low MTA3 expression.


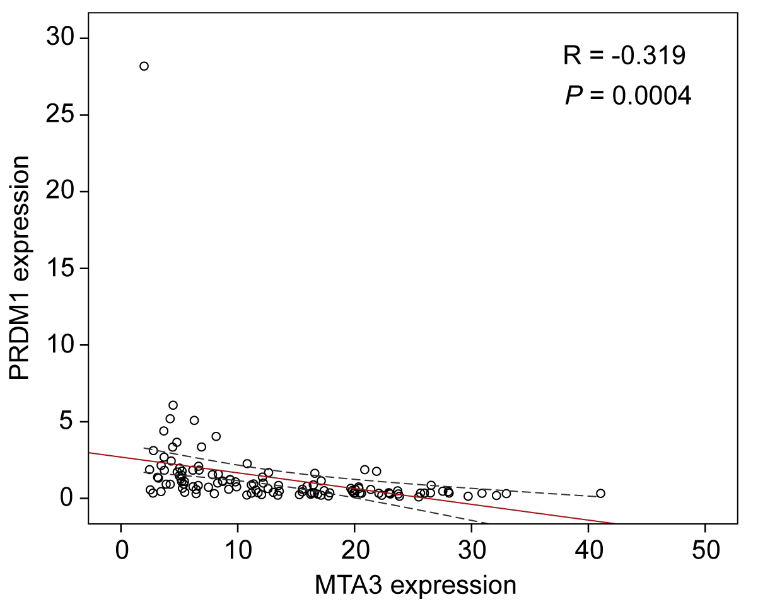


**Fig. S11.** MTA3 is inversely related to PRDM1 in TETs. Correlation analysis of MTA3 and PRDM1 expression in TCGA-TETs.


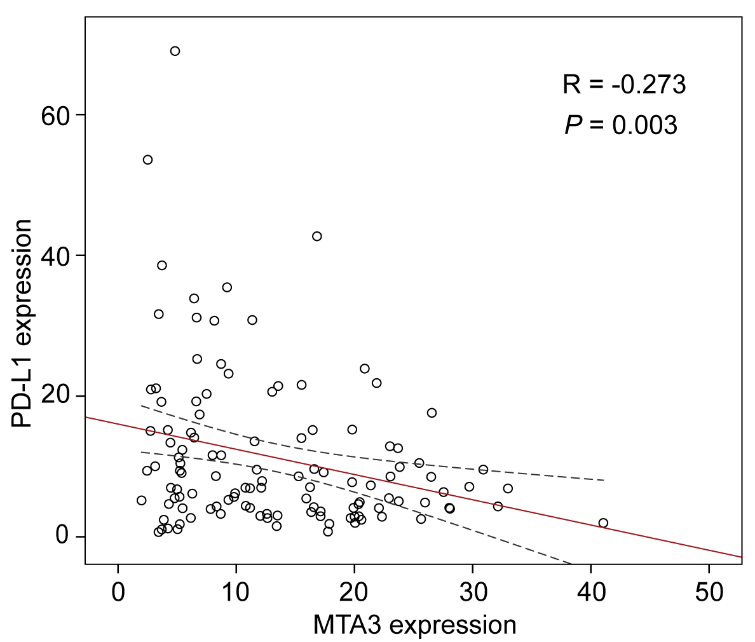


**Fig. S12.** MTA3 is inversely related to PD-L1 in TETs. Correlation analysis of MTA3 and PD-L1 expression in TCGA-TETs.
